# DEVELOPMENTAL ORIGINS OF EXCEPTIONAL HEALTH AND SURVIVAL: A FOUR-GENERATION COHORT STUDY

**DOI:** 10.1093/geroni/igad104.1613

**Published:** 2023-12-21

**Authors:** Matthew Keys, Pernille Larsen, Dorthe Pedersen, Alexander Kulminski, Mary Feitosa, Mary Wojczynski, Kaare Christensen

**Affiliations:** University of Southern Denmark, Odense, Syddanmark, Denmark; University of Southern Denmark, Odense, Syddanmark, Denmark; University of Southern Denmark, Odense, Syddanmark, Denmark; Duke University, Durham, North Carolina, United States; Washington University School of Medicine, St. Louis, Missouri, United States; Washington University School of Medicine, St. Louis, Missouri, United States; University of Southern Denmark, Odense, Syddanmark, Denmark

## Abstract

Little is known about the early life health trajectories associated with longevity, due to the length of follow-up required for such studies. In the Long Life Family Study (LLFS) we have identified 659 Danish long-lived sibships and their descendants across three generations and followed them through national civil and health registries. We have previously shown lower adult mortality and morbidity in both offspring and grandchildren generations. Here we compared perinatal, infant, and maternal health outcomes of third-generation grandchildren (n = 5637) and also fourth-generation great-grandchildren (14,908) born into these long-lived families between 1973-2018, to matched controls from the general population. Hazard ratios (HRs) and odds ratios (ORs) were estimated by Cox proportional hazards and conditional logistic regression models. A general pattern of reduced risk was observed in the grandchildren across a range of perinatal and infant health outcomes, most notably in infant mortality (HR = 0.53, 95% CI [0.36, 0.77]), preterm birth (OR = 0.82, [0.72, 0.93]), and low birth weight (OR = 0.83, [0.76, 0.90]). These associations were robust to further adjustment for parental education levels. Negligible infant survival advantage was observed in the great-grandchildren (HR = 0.90, [0.70, 1.17]). However, there were signals of benefit across some other perinatal outcomes, although much fewer and weaker in magnitude than in the grandchildren. Our findings suggest that phenotypic longevity may have developmental origins as early as the perinatal period, and that this effect persists over at least three generations.

